# The association between leukocyte telomere length and chronic obstructive pulmonary disease is partially mediated by inflammation: a meta-analysis and population-based mediation study

**DOI:** 10.1186/s12890-022-02114-8

**Published:** 2022-08-20

**Authors:** Tieshan Wang, Zhaoqi Jia, Sen Li, Yuxin Li, Tingting Yu, Tao Lu, Yuanyuan Shi

**Affiliations:** 1 Beijing Research Institute of Chinese Medicine, Beijing University of Chinese Medicine, Beijing, China; 2 School of Life Sciences, Beijing University of Chinese Medicine, Beijing, China; 3 Shenzhen Research Institute, Beijing University of Chinese Medicine, Shenzhen, China

**Keywords:** Telomere length, COPD, Inflammation, Mediation study, NHANES

## Abstract

**Background:**

Chronic obstructive pulmonary disease (COPD) is one of the major health issues worldwide. Pathophysiological changes in COPD are mainly reflected in the deterioration of lung function with aging.

**Methods:**

Considering that telomere length is a hallmark of biological aging, we first performed a meta-analysis to summarize the current knowledge about the relationship between telomere length and COPD and then employed individual-level data from the continuous National Health and Nutrition Examination Survey (NHANES) to investigate whether telomere length could reflect accelerated aging in COPD and serve as an independent predictor. A mediation study was further performed to examine whether the association between telomeres and COPD could be mediated by inflammation, as one of the most important etiologies and characteristics of COPD.

**Results:**

The four studies included in our meta-analysis were with high heterogeneity (I^2^ = 95.7%, *P*_het_ < 0.001), and the pooled relative risk for COPD comparing the shortest tertile versus the longest tertile was 4.06 (95% CI = 1.38 to 11.96). Of the 6,378 subjects in the individual-level data analyses using NHANES, 455 were diagnosed with COPD, and multivariable-adjusted logistic regression also indicated that short telomere length was associated with COPD. Consistently, cubic regression spline analyses showed that long telomeres exhibited a significant association with a decreased risk of COPD. In the subsequent mediation analyses, C-reactive protein concentration, white blood cells count and blood neutrophil count, as inflammatory biomarkers, showed a significant indirect effect on the relationship between telomere length and COPD.

**Conclusion:**

Accelerated aging in COPD could be characterized by excessive telomere shortening, and inflammatory response might be involved in the underlying mechanisms of COPD pathogenesis promoted by short telomere length. Telomere length measurement may facilitate clinical translational research and targeted therapy of COPD.

**Supplementary Information:**

The online version contains supplementary material available at 10.1186/s12890-022-02114-8.

## Introduction

Chronic obstructive pulmonary disease (COPD) is becoming an important health issue worldwide due to their protracted and refractory pathologic processes [1]. It can be induced or aggravated by numerous pathogenic factors, such as chronic lower respiratory tract inflammation and long-term inhalation of harmful substances [2], and can lead to a series of pathological features, such as mucus cell proliferation, airway inflammation, and lung parenchymal injury [3].

Although the pathogenesis of COPD remains unclear, extensive evidence indicates a close association between COPD and age [3]. For example, the incidence of COPD increases dramatically after age 40 [4], and the physiological aging of the lung is represented by the reduction of the surface area of gas exchange caused by the expansion of alveoli, which leads to a reduction in the lung static elastic recovery and an increase in functional residual capacity [5]. Telomeres, comprised of TTAGGG repeats, act as DNA caps, which prevent the degeneration or remodeling of chromosomes [6]. Telomeres become shorter with cell replication; thus, the lifespan represented by telomeres is biological, rather than chronological [4]. Indeed, the association between telomere length and disease progression could be independent of the chronological age of the organism [4]. As a result, leukocyte telomere length has been employed as a biomarker of aging [7], which improves the treatment method and therapeutic effect of targeted therapy [8]. Given that excessive telomere attrition has been found in COPD patients [9], we first performed a meta-analysis to summarize the current knowledge about the relationship between telomere length and COPD and then employed individual-level data from the NHANES to examine whether telomere length could reflect accelerated aging in COPD and serve as an independent predictor. As one of the most important etiologies and characteristics of COPD, inflammation has been known to be strongly associated with both telomere length and COPD [10]. Thus, a mediation study was further applied to test whether inflammation acts as a potential mediator in the relationship between telomeres and COPD.

## Methods

### Meta-analysis

**Fig. 1:**
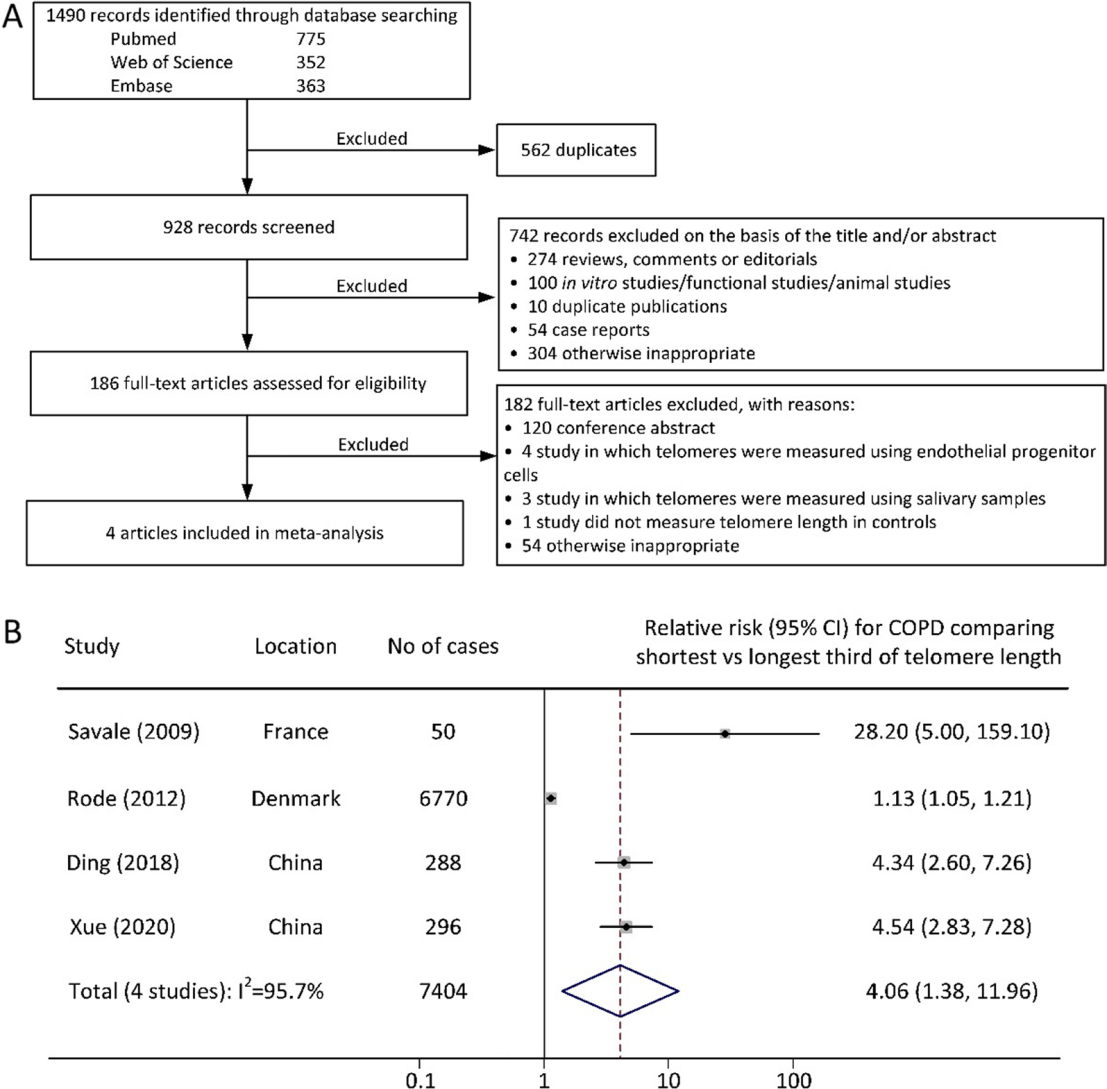
Study flow diagram (**A**) and forest plot (**B**) of the meta-analysis

We employed a structured strategy to search three electronic databases, PubMed, Web of Science and EMBASE, for studies published up to February 2020 using the following key terms: ‘telomere’, ‘telomeric’, ‘telomerase’, ‘T/S ratio’, ‘lung disease’, ‘chronic obstructive’, ‘pulmonary disease’, ‘airflow obstruction’, ‘emphysema’, ‘bronchitis’, ‘COPD’, ‘AECOPD’, ‘COAD’, ‘COBD’, ‘AECB’, ‘forced expiratory volume’ and ‘vital capacity’. The inclusion and exclusion criteria and search results are outlined in a flow diagram (Fig. 1A). Two authors independently assessed the titles and abstracts based on the a priori selection criteria to ensure that articles were eligible. The full texts of papers that matched the inclusion criteria were retrieved and screened for final inclusion. In all the meta-analysis populations, fixed forced expiratory volume in 1 s (FEV1)/forced vital capacity (FVC) ratio < 70% was the criteria for COPD definition, as suggested by the Global initiative for chronic Obstructive Lung Disease (GOLD). We extracted the characteristics of the studies, including first author, location, population, telomere length assay and odds ratios (ORs). Next, we converted the effect sizes of the included studies to common metrics; that is, these studies were compared using the shortest versus longest one-third of telomere length. Relative risk was transformed by assuming the approximately normal distribution of telomere length and the log-linear relationship between telomere length and COPD. The effect size for the longest versus shortest one-fourth was calculated as 1.37 times the longest versus the shortest one-third, and the effect size of the bottom versus the top one-half was calculated as 0.86 times the longest versus shortest third. Heterogeneity of studies was assessed by I^2^. Stata 12.0 software was used for analysis in this study.

### Individual-level data analyses using NHANES

The NHANES collects information about the health condition of the noninstitutional civilian population in the U.S. [11–14]. Data from NHANES (1999–2002) (n = 6,378) were employed to investigate the association between telomere length and COPD (Additional file 1: Figure S1). For the analyses involving smoking history adjustment, participants with missing data were further excluded (Additional file 1: Tables S1–S4). Participants with missing information on inflammatory biomarkers were excluded from the mediation study, and 4,011 adults were included in the analyses. Blood samples were collected from eligible participants and stored for DNA analyses. The telomere length was recorded as telomere length/standard reference DNA (T/S ratio) [15]. COPD was defined by a self-reported doctor diagnosis of chronic bronchitis or emphysema as previously described [16, 17], and the relationship between telomere length and COPD status was adjusted for various potential confounding factors as described previously [14, 18]. Blood samples were collected and tested in accordance with standard procedures to obtain the concentrations of five inflammatory biomarkers [C-reactive protein (CRP), fibrinogen, white blood cells (WBC) count, blood neutrophil count (B-Neu) and blood eosinophil count (B-Eos)] employed in the mediation analysis. The association between telomere length and COPD status was studied by logistic regression to calculate odds ratios and 95% confidence intervals (CIs). *P*_trend_ was also calculated for telomere length quartiles (first quartile = 5.31, second quartile/median = 5.68 and third quartile = 6.10 kbp). SAS 9.4 software (SAS Institute Inc., Cary, NC) was employed to perform statistical analyses. Correlation analysis between chronological age and telomere length was performed in R using the PerformanceAnalytics package. In the mediation analyses, the telomere length, inflammatory biomarkers, and COPD status were selected as the independent variable (X), mediator (M) and dependent variable (Y), respectively. The total effect of X on Y was decomposed into a direct effect (i.e., an effect of X on Y controlling for M) and an indirect effect (i.e., an effect of X on Y mediated by M) [19], which were calculated using the PROCESS macro developed by Hayes [20]. All types of inflammatory biomarkers were log-transformed, and age, sex, education, race, poverty income ratio, body mass index, smoking status, and alcohol-use status were adjusted as covariates. Full methods of individual-level data analyses using NHANES can be found in Additional file 1.

## Results

The studies included in our meta-analysis involved 7,404 COPD cases, and were conducted in China or Europe. All the studies indicated that shorter telomere length was associated with a higher risk of COPD (Fig. 1B). Because of the high heterogeneity among studies (I^2^ = 95.7%, *P*_het_ < 0.001), we performed a random-effect meta-analysis, and the pooled relative risk for COPD comparing the shortest tertile versus the longest tertile was 4.06 (95% CI = 1.38 to 11.96). It is also of note that the high heterogeneity was presumably caused by the different sample sizes and study designs between the Rode et al. study (cross-sectional design) and the others (case–control designs), because a reduced heterogeneity (I^2^ = 52.7%, *P* = 0.121) was observed after excluding this study. Furthermore, excluding Savale et al. study led to a reduced relative risk for COPD comparing the shortest tertile versus the longest tertile (2.76; 95% CI = 0.92 to 8.26).

Because of the small number of studies and their significant heterogeneity in the meta-analysis, we next investigated the potential association between leukocyte telomere length and COPD using individual-level data from the NHANES, which represents the noninstitutional U.S. population. We first examined the correlation between chronological age and telomere length, and Additional file 1: Figure S2 shows that telomere length was negatively correlated with chronological age in the study population and sex-specific population (*P* < 0.01). The linear regression model suggested that a 1-year increase in age was correlated with telomere length decreases of 14.75 ± 0.80, 15.49 ± 0.75 and 14.17 ± 1.10 bp in the overall, male and female populations, respectively. Demographics of the study population by telomere length quartiles are provided in Table 1. Compared to participants with a long telomere length (quartile 4), participants with a short telomere length (quartile 1) had higher chance to be ≥ 40 years old, be white, have less education, be overweight, be a nondrinker, have a history of diabetes and hypertension and be less physically active. Moreover, telomere length was correlated with COPD (*P* < 0.01) but not asthma (*P* = 0.38). In addition, our results suggested that COPD was correlated with sex, age, race, education level, PIR, smoking status, hypertension history and physical activity status (Table 2).

**Table 1:** Weighted characteristics of the study population by telomere length quartiles-NHANES 1999–2002

Variable	Status	Overall			Telomere length												*P* value
		N	%	s.e.	Quartile 1			Quartile 2			Quartile 3			Quartile 4			
					N	%	s.e.	N	%	s.e.	N	%	s.e.	N	%	s.e.	
Gender	Male	3102	49.19	0.63	861	50.54	1.29	807	50.57	1.36	720	47.03	1.51	714	48.95	1.33	0.26
	Female	3276	50.81	0.63	734	49.46	1.29	787	49.43	1.36	875	52.97	1.51	880	51.05	1.33	
Age	20–39 years	2322	40.02	1.13	232	18.22	1.51	475	32.71	1.98	690	44.75	2.24	925	58.40	2.60	< 0.01
	40–59 years	1976	38.72	0.90	427	38.00	2.12	549	43.62	1.69	535	40.03	1.95	465	33.74	1.90	
	60–79 years	1666	17.97	0.69	694	34.82	1.85	466	20.17	1.55	321	13.82	1.16	185	7.27	1.00	
	> 80 years	414	3.28	0.27	242	8.96	0.78	104	3.49	0.53	49	1.40	0.29	19	0.60	0.18	
Race	White	3346	74.64	1.76	893	79.00	2.78	846	76.12	2.06	810	73.61	2.16	797	71.02	2.37	< 0.01
	Black	1033	8.71	1.00	209	6.67	0.95	237	7.62	1.30	243	8.07	0.90	344	11.80	1.58	
	Others	1999	16.65	2.08	493	14.33	2.93	511	16.26	2.14	542	18.32	2.30	453	17.19	2.57	
Education	< high school	2004	19.58	1.06	626	25.37	1.73	505	19.74	1.50	476	19.36	1.49	397	15.32	1.09	< 0.01
	= high school	1483	26.01	1.06	367	26.94	1.33	361	26.32	1.67	350	24.33	1.86	405	26.61	1.68	
	> high school	2891	54.40	1.68	602	47.70	1.85	728	53.94	2.15	769	56.31	2.78	792	58.07	1.89	
Poverty income ratio	< 1	1074	13.09	0.96	268	11.54	1.23	260	12.07	1.42	261	12.65	1.52	285	15.56	1.44	0.14
	1 ≤ PIR ≤ median	2659	35.87	1.40	714	38.42	2.03	663	36.48	1.58	647	34.07	2.21	635	35.07	2.07	
	> median	2645	51.04	1.99	613	50.04	2.45	671	51.45	2.51	687	53.28	2.73	674	49.36	2.58	
Body mass index	≥ 25	4380	65.30	0.89	1157	71.54	1.38	1102	66.56	1.78	1102	64.69	1.74	1019	60.09	1.74	< 0.01
Smoking	Yes	3118	50.27	1.41	843	53.74	2.11	791	51.37	1.81	761	49.46	1.46	723	47.45	2.63	0.07
Alcohol use	Yes	4374	73.32	2.08	1050	68.65	2.10	1089	72.41	2.56	1114	75.01	2.20	1121	76.07	3.14	0.02
Diabetes	Yes	590	6.36	0.39	223	10.29	0.86	158	6.81	0.90	118	4.96	0.69	91	4.33	0.62	< 0.01
High blood pressure	Yes	1877	25.30	0.91	604	34.45	1.74	505	26.55	1.54	437	24.51	1.54	331	18.09	1.24	< 0.01
Physical activity	Yes	3639	64.59	1.51	807	58.25	2.01	890	62.44	2.44	941	66.65	1.68	1001	69.32	1.94	< 0.01
COPD	Yes	455	7.89	0.46	162	11.78	0.92	116	8.82	0.87	95	6.02	0.77	82	5.88	0.52	< 0.01
Asthma	Yes	656	11.62	0.54	149	11.49	0.96	172	12.93	1.04	165	10.54	0.95	170	11.54	1.07	0.38

*N* Number; %, weighted percent; *s.e.* standard error; *NHANES* National Health and Nutrition Examination Survey

*P* values were calculated from Rao-Scott Chi-square tests

**Table 2:** Weighted characteristics of the study population by COPD status-NHANES 1999–2002

Variable	Status	COPD						*P* value
		Yes			No			
		N	%	s.e.	N	%	s.e.	
Gender	Male	171	33.68	3.10	2931	50.51	0.61	< 0.01
	Female	284	66.32	3.10	2992	49.49	0.61	
Age	20–39 years	122	32.56	2.19	2200	40.66	1.20	< 0.01
	40–59 years	132	35.46	2.94	1844	39.00	0.95	
	60–79 years	161	27.16	2.60	1505	17.19	0.67	
	> 80 years	40	4.82	0.97	374	3.15	0.24	
Race	White	295	81.11	2.63	3051	74.08	1.79	0.01
	Black	59	6.42	1.29	974	8.91	1.01	
	Others	101	12.47	2.93	1898	17.01	2.07	
Education	< high school	155	26.59	2.31	1849	18.98	1.04	< 0.01
	= high school	118	30.88	2.74	1365	25.60	1.15	
	> high school	182	42.53	2.83	2709	55.42	1.74	
Poverty income ratio	< 1	94	20.00	3.64	980	12.50	0.85	< 0.01
	1 ≤ PIR ≤ median	201	42.66	3.11	2458	35.29	1.39	
	> median	160	37.34	3.78	2485	52.21	1.93	
Body mass index	≥ 25	308	65.51	2.45	4072	65.28	0.98	0.94
Smoking	Yes	290	64.98	2.52	2828	49.01	1.43	< 0.01
Alcohol use	Yes	306	71.49	2.58	4068	73.48	2.15	0.42
Diabetes	Yes	50	8.22	1.29	540	6.20	0.41	0.10
High blood pressure	Yes	192	37.11	2.68	1685	24.29	0.90	< 0.01
Physical activity	Yes	226	53.75	4.11	3413	65.52	1.46	< 0.01

*N* Number; %, weighted percent; *s.e.* standard error; *NHANES* National Health and Nutrition Examination Survey

*P* values were calculated from Rao-Scott Chi-square tests

**Fig. 2:**
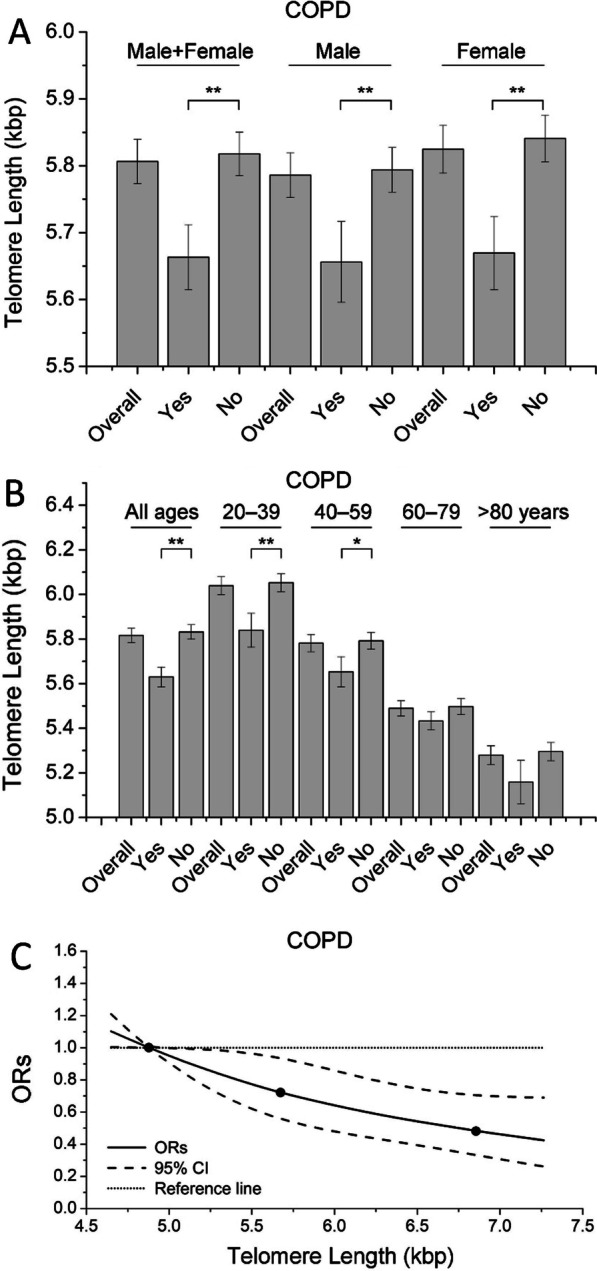
**A** Telomere lengths of participants according to COPD status, stratified by sex. **B** Telomere lengths of participants according to COPD status, stratified by age. **C** Adjusted dose–response association between telomere length and risk for COPD. Telomere length was coded using an RCS function with three knots (dots in black) located at the 5th, 50th and 95th percentiles of its distribution. The *Y*-axis represents the adjusted odds ratio for COPD for any value of telomere length compared to a referent level of the 5th percentile of its distribution (NHANES 1999–2002). *denotes *P* < 0.05 and **denotes *P* < 0.01

ORs from logistic regression analysis for COPD were then calculated in overall and sex-specific populations by employing telomere length quartiles as an independent variable (Table 3). Both crude and confounder-adjusted logistic regression indicated that telomere length was associated with COPD (*P*_trend_ < 0.05). For COPD, a multivariable-adjusted model showed that participants with a short telomere length (quartile 1) exhibited an OR of 1.68 (95% CI = 1.19–2.37) compared to subjects with a long telomere length (quartile 4). Considering that smoking is one of the most profound risk factors for COPD, smoking history (pack-years) was also calculated. The results suggested that adults with a short telomere length (quartile 1) were more likely to have > 20 pack-years than participants with a long telomere length (quartile 4) (Additional file 1: Table S1). Results from linear regressions indicated that both smoking status (*P* = 0.03) and smoking history (*P* < 0.01) were associated with telomere length. Smoking history was also correlated with COPD (*P* < 0.01) (Additional file 1: Table S2), and increased pack-year smoking was associated with an elevated risk of COPD (*P*_trend_ < 0.01) (Additional file 1: Table S3). More importantly, an increased risk of COPD in subjects with a short telomere length was consistently observed after adjusting for smoking history as a confounder in multivariate logistic regression analysis (Additional file 1: Table S4). Consistent with these results, COPD patients had significantly shorter age-adjusted telomere lengths than non-COPD participants (Fig. 2A). The age-stratified analysis revealed a generally reduced telomere length in subjects with COPD, but the difference was not statistically significant in participants aged 60 years and older (Fig. 2B). We next treated telomere length as a continuous variable to study the dose–response relationship between telomere length and COPD status using a three-knot cubic regression spline (Fig. 2C), which permits a flexible and not inherently linear shape of the relationship between the exposure and the outcome. In Fig. 2C, the Y-axis represents covariate-adjusted ORs to present COPD for any value of telomere length compared to subjects with a 4.88-kbp telomere length (5th percentile of the telomere length distribution as a referent level). Spline analysis indicated that the ORs of COPD generally decreased with telomere length, and the upper bound of the 95% CI exceeded the reference line (OR = 1) at a telomere length of approximately 4.98 kbp.

**Table 3:** Odds ratio for COPD by telomere length quartiles-NHANES 1999–2002

Telomere length	COPD		
	N	cOR (95% CI)	aOR (95% CI)^a^
Male + Female^b^	455		
Q4	82	1.00	1.00
Q3	95	1.03 (0.72–1.45)	0.95 (0.66–1.36)
Q2	116	1.55 (1.19–2.01)	1.35 (0.99–1.86)
Q1	162	2.14 (1.67–2.74)	1.68 (1.19–2.37)
*P* for trend		< 0.01	< 0.01
Male	171		
Q4	27	1.00	1.00
Q3	30	0.97 (0.48–1.95)	0.90 (0.45–1.81)
Q2	45	1.37 (0.87–2.16)	1.28 (0.77–2.14)
Q1	69	2.11 (1.41–3.15)	1.72 (0.98–3.00)
*P* for trend		< 0.01	0.04
Female	284		
Q4	55	1.00	1.00
Q3	65	1.04 (0.69–1.57)	0.95 (0.62–1.45)
Q2	71	1.69 (1.20–2.38)	1.41 (0.95–2.12)
Q1	93	2.21 (1.55–3.14)	1.64 (1.07–2.51)
*P* for trend		< 0.01	< 0.01

*cOR* Crude odds ratio; *aOR* Adjusted odds ratio; *CI* confidence interval; *N* Number; *NHANES* National Health and Nutrition Examination Survey

^a^Model was adjusted for age, age square, education, race, poverty income ratio, body mass index, smoking status, alcohol-use status, diabetes status, high blood pressure status and physical activity status

^b^Sex was further adjusted

The mediation analysis showed that participants with a shorter telomere length corresponded to significantly higher values of CRP (*P* < 0.001), WBC count (*P* < 0.001) and B-Neu (*P* = 0.016) (X → M). Almost every inflammatory factor (CRP, fibrinogen, WBC count and B-Neu) involved in this study showed a significant positive association with COPD (*P* < 0.01), except B-Eos (*P* = 0.148), after controlling for the effects of telomere length on COPD (M|X → Y). Telomere length was negatively associated with COPD (X|M → Y), which is consistent with the previous analysis. CRP, WBC count and B-Neu significantly mediated the association between telomere length and COPD, as the 95% bootstrap CI did not include the null (X → M → Y) (Table 4), and the proportions mediated for these three inflammatory biomarkers were 8.6%, 6.2% and 4.2%, respectively. Interestingly, CRP and B-Neu also showed significant indirect effect on the relationship between age and COPD (Additional file 1: Table S5).

**Table 4:** Mediation of 5 inflammatory factors for the associations between telomere length and COPD

	M:CRP	*P* value	M: Fibrinogen	*P* value	M:WBC	*P* value	M:B-Neu	*P* value	M:B-Eos	*P* value
	β(95%CI)		β(95%CI)		β(95%CI)		β(95%CI)		β(95%CI)	
X → M	−0.337 (−0.490, −0.183)	< 0.001	0.006 (−0.022, 0.033)	0.696	−0.069 (−0.106, −0.032)	< 0.001	−0.060 (−0.109, −0.011)	0.016	−0.049 (−0.128, 0.031)	0.232
M|X → Y	0.193 (0.090, 0.296)	< 0.001	0.941 (0.367, 1.516)	0.001	0.670 (0.242, 1.099)	0.002	0.532 (0.200, 0.864)	0.002	0.152 (−0.054, 0.358)	0.148
X|M → Y	−0.692 (−1.251, −0.133)	0.015	−0.767 (−1.326, −0.209)	0.007	−0.700 (−1.260, −0.141)	0.014	−0.723 (−1.283, −0.163)	0.011	−0.763 (−1.335, −0.191)	0.009
X → M → Y	−0.065 (−0.124, −0.026)	–	0.005 (−0.021, 0.036)	–	−0.046 (−0.094, −0.017)	–	−0.032 (−0.077, −0.007)	–	−0.007 (−0.038, 0.003)	–

X = telomere length; M = mediator (log-transformed CRP, fibrinogen, WBC, B-Neu or B-Eos); Y = COPD

Analytical process includes: 1) an effect of X on M (X → M); 2) an effect of M on Y controlling for X (M|X → Y); 3) a direct effect of X on Y, i.e. an effect of X on Y controlling for M (X|M → Y); and 4) an indirect effect of X on Y, i.e. an effect of X on Y mediated by M (X → M → Y). Each process was adjusted for age, sex, education, race, poverty income ratio, body mass index, smoking status, alcohol-use status

## Discussion

Telomeres at the ends of chromosomes become shorter with cell replication, suggesting a link between telomere length and cellular senescence [4, 21, 22]; thus, telomere length is widely believed to represent the biological age of eukaryotes [8]. Because functional lung alterations due to aging enhance the susceptibility to COPD [23], we examined whether telomere length could predict COPD by summarizing the relevant literature through meta-analysis, which indicated an increased relative risk of COPD with a shorter telomere length. Moreover, our analyses using individual-level data indicated telomere length was significantly shorter in COPD patients, and CRP, WBC count and B-Neu, as inflammatory markers, showed a significant indirect effect on the association between telomere length and COPD, suggesting that inflammation partially mediated this association.

The association between telomere length and COPD has been illustrated by several epidemiological studies. A prospective Danish study revealed a relationship between COPD and short telomere length [24]. Another French study reported shorter telomeres in peripheral WBCs or lung cells in patients with COPD than in healthy controls [25]. Moreover, it is known that patients with emphysema may have shorter telomere lengths in certain cell types (e.g., peripheral blood lymphocytes, fibroblasts) [26]. Thus, the results from our individual-level data analyses using NHANES data are consistent with epidemiological studies showing that accelerated aging in COPD can be characterized by excessive telomere shortening.

The positive role of short telomere length in promoting the development of COPD is evidenced in short telomere syndromes, a group of genetic disorders induced by mutations in telomere maintenance-related genes [8]. As one of the most common complications of short telomere syndrome, lung diseases, such as emphysema, tend to induce morbidity and mortality [27]. Notably, telomere length but not telomerase loss is deemed a degenerative genetic defect because no degenerative phenotype is observed in telomerase-null mice with normal telomere lengths [28, 29]. This is consistent with the situation in humans in which lung disease onset is correlated with the degree of telomere shortening in patients with mutations in telomerase and telomere maintenance genes [8]. Thus, evidence from short telomere syndromes reveals that short telomere length plays an important role in the development of COPD. As suggested by Fletcher–Peto curve, an accelerated decline of FEV1 can be observed during aging, and patients with COPD have FEV1 decline that occurs more quickly compared to controls [30]. However, other studies also indicate the accelerated decline in FEV1 is not a prerequisite of COPD occurrence, and the baseline lung function or the patterns of lung function changes are important to the development of COPD [31, 32]. Understanding the independent and interactive role of accelerated aging and lung function trajectories is important to identify individuals with COPD in early adulthood, which warrants further investigation.

Smoking is a well-known risk factor for COPD, and oxidative stress mediated by smoking promotes cell turnover, causes DNA damage and induces dysregulation of protease and antiprotease balance, leading to emphysema-like pathological changes in the lung [4]. Smoking also reduces telomere length in a dose–effect relationship [33]. For instance, women who smoke can accelerate the loss of telomeres from circulating lymphocytes, and each pack of cigarettes a year can cause 5 bp of telomere shortening [34]. Our results consistently demonstrated that smoking history was correlated with telomere length and COPD status in multivariate logistic regression. One underlying mechanism of smoking-mediated telomere attrition is that smoking accelerates the acetylation and degradation of shelterin TPP1 by disrupting the interaction between TPP1 and the SIRT1 complex [35]. In addition to promoting telomere shortening in the lung epithelium, smoking can also induce dysregulation of p21, which mediates cellular senescence [36]. The oxidative stress induced by smoking is also correlated with low-grade systemic inflammation [33], and SIRTs may serve as a target for anti-inflammatory and anti-aging processes [37]. In the lungs of patients with COPD, downregulation of SIRT1 is due to smoking-mediated posttranslational oxidative modification [4, 37]. In animal experiments, SIRT1 levels in the lungs of rats can also be reduced by cigarette smoke, indirectly leading to an increase in inflammatory cytokines [38]. In summary, smoking promotes telomere shortening, cellular senescence and inflammation in COPD.

The cell senescence hypothesis associated with short telomere length is widely recognized as one of the biological mechanisms of COPD. Cell division shortens telomere length, and it hypothesizes that a telomere length below a threshold leads to cellular senescence, a state in which cells lose the ability to divide [39]. Indeed, the promoted cellular senescence of both alveolar epithelial and endothelial cells has been found in emphysema patients [26]. A study of the mechanism of cellular senescence-mediated lung disease indicated that critically short telomeres symbolized a DNA damage response that activated cellular apoptosis [40]. To compensate for such alveolar cell apoptosis, a high level of alveolar cell proliferation is found in the emphysematous lung [41, 42]. Like other somatic cells, excessive proliferation eventually induces senescence of alveolar cells, and apoptotic cells are no longer replaced, which in turn contributes to the loss of alveolar structure and reduction of lung surface area [26]. Cellular senescence of mesenchymal precursor cells may also be a cause of connective tissue defects in patients with COPD, a physiological change that promotes the production of extracellular matrix proteins and affects signaling pathways involved in alveolar modulation [43]. Malfunction of the shelterin complex that protects telomeres from DNA repair mechanisms can promote telomere dysfunction and result in cellular senescence [35]. For example, a reduced level of shelterin telomere protection protein 1 leads to SIRT1-mediated telomere attrition and subsequent cellular senescence in COPD [35]. Furthermore, in type 2 alveolar epithelial cells (AEC2s), the deletion of the shelterin component telomeric repeat binding factor 2 (TERF2) is associated with DNA damage, which leads to cell senescence and lung remodeling [44]. In conclusion, cellular aging, which short telomeres can represent, has been linked to decreased lung function [39]. Understanding the biological mechanisms of lung aging will be helpful for the treatment of lung diseases, such COPD and pulmonary fibrosis [45]. For example, telomerase can be activated by adeno-associated serotype 9 vector (AAV9)–Tert gene therapy, which has therapeutic effect on lung fibrosis in a mouse model [46]. Thus, telomere shortening may serve as a promising target in the development of novel strategies to treat lung diseases.

An increased level inflammation can be observed during aging, which is called “inflammaging” [47]. As an important marker of inflammation, CRP is synthesized in liver upon stimulation of proinflammatory cytokines, and elderly adults tend to have a higher level of Serum CRP [48]. Patients with COPD also have an elevated CRP level [49], and CRP can independently predict the prognosis of COPD in participants with airway obstruction [50]. These established links between CRP and aging/COPD are further supported by our observation that CRP partially mediated the association between telomere length and COPD. At molecular level, the hyperactivity of transcription factor NF-κB in senescent cells with short telomere lengths contributes to the generation of inflammation [51, 52], and high levels of circulating or local proinflammatory cytokines are pathologic features of COPD [3]. The autoimmune phenotype may explain the persistent inflammation in COPD due to an increase in the number of circulating senescent T cells in patients [53, 54]. Alveolar senescence and mesenchymal progenitor cell dysfunction are also thought to be involved in inflammatory and autoimmune processes in COPD [55–57]. Furthermore, the immune response caused by senescent AEC2s with telomere dysfunction can provoke inflammation in the lungs, which is related to the upregulation of cytokine signaling pathways [44]. Bone marrow-derived mesenchymal stem cells (MSCs) inhibit the release of proinflammatory factors and stimulate the activity of T lymphocytes [58, 59]. However, MSCs in COPD are impaired in function, which weakens the suppression of proinflammatory cytokines, leading to contractile bronchitis [43]. Clinical evidence suggests that the plasma content of numerous cytokines is increased in COPD patients, and interleukin-6 in COPD patients is inversely proportional to telomere length [25]. In sum, the current study supported the notion that aging caused by shortening telomeres is naturally associated with increased levels of proinflammatory cytokines that promote the development of COPD by the inflammatory response [44].

This study has several limitations. First, causality of the associations observed in this study could not be tested because of its cross-sectional design, and thus reverse causality could not be prevented. For example, it’s impossible to test whether inflammation leads to short telomeres length, which is required by the assumption of mediation analyses (i.e., no reciprocal causation between the exposure and the mediator). Second, information about COPD status of participants was self-reported, and it is more accurate to define COPD by spirometry data. Third, replication of our findings from the analyses using NHANES data in large and independent cohorts is needed. Despite these limitations, the current study has several strengths. Numerous demographic and inflammatory biomarkers have been collected by NHANES, allowing us to adjust the potential confounders in the mediation analyses. Moreover, the sample for NHANES is selected to represent the noninstitutionalized civilian residents the United States. Furthermore, multiple data analysis methods, such as meta-analysis and mediation analysis, were employed in our study to illustrate the association of telomere length shortening with COPD and the mediating role of inflammation in this association.

## Conclusions

Both meta-analysis and individual-level data analysis indicate an elevated risk of COPD in participants with a shorter telomere length, suggesting that excessive telomere shortening in peripheral circulation could serve as a biomarker to characterize accelerated aging in COPD. Possible underlying mechanisms of COPD pathogenesis promoted by short telomere length may involve an inflammatory response in the lung, although smoking is the major etiological factor. More fundamental research on the pathogenesis of COPD is required to understand the etiology of such multifactorial systemic diseases and provide novel diagnostic, prognostic, and therapeutic approaches.

## Supplementary Information

**Additional file 1.** **Supplementary Figure 1.** Participant enrollment flowchart including the exclusion criteria-NHANES 1999-2002. **Supplementary Figure 2.** Correlation between chronological age and telomere length. Three data points with telomere length > 10 kbp were removed to provide a better view of the scatter plots, and age above 85 was top coded as 85 to reduce the risk of disclosing the identity of participants in the NHANES 1999-2002. **Supplementary Table 1**. Weighted smoking history of the study population by telomere length quartiles (n=6014)-NHANES 1999-2002. **Supplementary Table 2**. Weighted smoking history of the study population by COPD status (n=6014)-NHANES 1999-2002. **Supplementary Table 3.** Odds ratio for COPD by smoking status (n=6378) and smoking history (n=6014)-NHANES 1999-2002. **Supplementary Table 4.** Odds ratio for COPD by telomere length quartiles (n=6014)-NHANES 1999-2002. **Supplementary Table 5.** Mediation of 5 inflammatory factors for the associations between age and COPD.

## Data Availability

The data employed in this study are from the NHANES 1999–2002, which are publicly available and can be downloaded from the NHANES website: http://www.cdc.gov/nchs/nhanes.htm.

## Abbreviations

NHANES: National Health and Nutrition Examination Survey
NCHS: National Centers for Health Statistics
PIR: Poverty income ratio
BMI: Body mass index
SE: Standard error
cOR: Crude odds ratio
aOR: Adjusted odds ratio
CI: Confidence interval
TERF2: Telomeric repeat-binding factor 2
AEC2s: Type 2 alveolar epithelial cells
MSCs: Mesenchymal stem cells
TPP1: Telomere protection protein 1
SIRT1: Sirtuin 1
SA β-gal: Senescence-associated β-galactosidase
