# Supplementary material for: The association between leukocyte telomere length and chronic obstructive pulmonary disease is partially mediated by inflammation: a meta-analysis and population-based mediation study
Source: BMC Pulm Med. 2022 Aug 20;22:320. doi: 10.1186/s12890-022-02114-8 (PMC9392327; doi:10.1186/s12890-022-02114-8)
Supplement: Supplementary file 1 — Additional file 1. Supplementary Figure 1. Participant enrollment flowchart including the exclusion criteria-NHANES 1999-2002. Supplementary Figure 2. Correlation between chronological age and telomere length. Three data points with telomere length > 10 kbp were removed to provide a better view of the scatter plots, and age above 85 was top coded as 85 to reduce the risk of disclosing the identity of participants in the NHANES 1999-2002. Supplementary Table 1. Weighted smoking history of the study population by telomere length quartiles (n=6014)-NHANES 1999-2002. Supplementary Table 2. Weighted smoking history of the study population by COPD status (n=6014)-NHANES 1999-2002. Supplementary Table 3. Odds ratio for COPD by smoking status (n=6378) and smoking history (n=6014)-NHANES 1999-2002. Supplementary Table 4. Odds ratio for COPD by telomere length quartiles (n=6014)-NHANES 1999-2002. Supplementary Table 5. Mediation of 5 inflammatory factors for the associations between age and COPD. [file 12890_2022_2114_MOESM1_ESM.docx]

**Supplementary Material**

Supplementary Figure 1. Participant enrollment flowchart including the exclusion criteria-NHANES 1999-2002.

Supplementary Figure 2. Correlation between chronological age and telomere length. Three data points with telomere length > 10 kbp were removed to provide a better view of the scatter plots, and age above 85 was top coded as 85 to reduce the risk of disclosing the identity of participants in the NHANES 1999-2002.

Supplementary Table 1. Weighted smoking history of the study population by telomere length quartiles (n=6014)-NHANES 1999-2002.

Supplementary Table 2. Weighted smoking history of the study population by COPD status (n=6014)-NHANES 1999-2002.

Supplementary Table 3. Odds ratio for COPD by smoking status (n=6378) and smoking history (n=6014)-NHANES 1999-2002.

Supplementary Table 4. Odds ratio for COPD by telomere length quartiles (n=6014)-NHANES 1999-2002.

Supplementary Table 5. Mediation of 5 inflammatory factors for the associations between age and COPD.


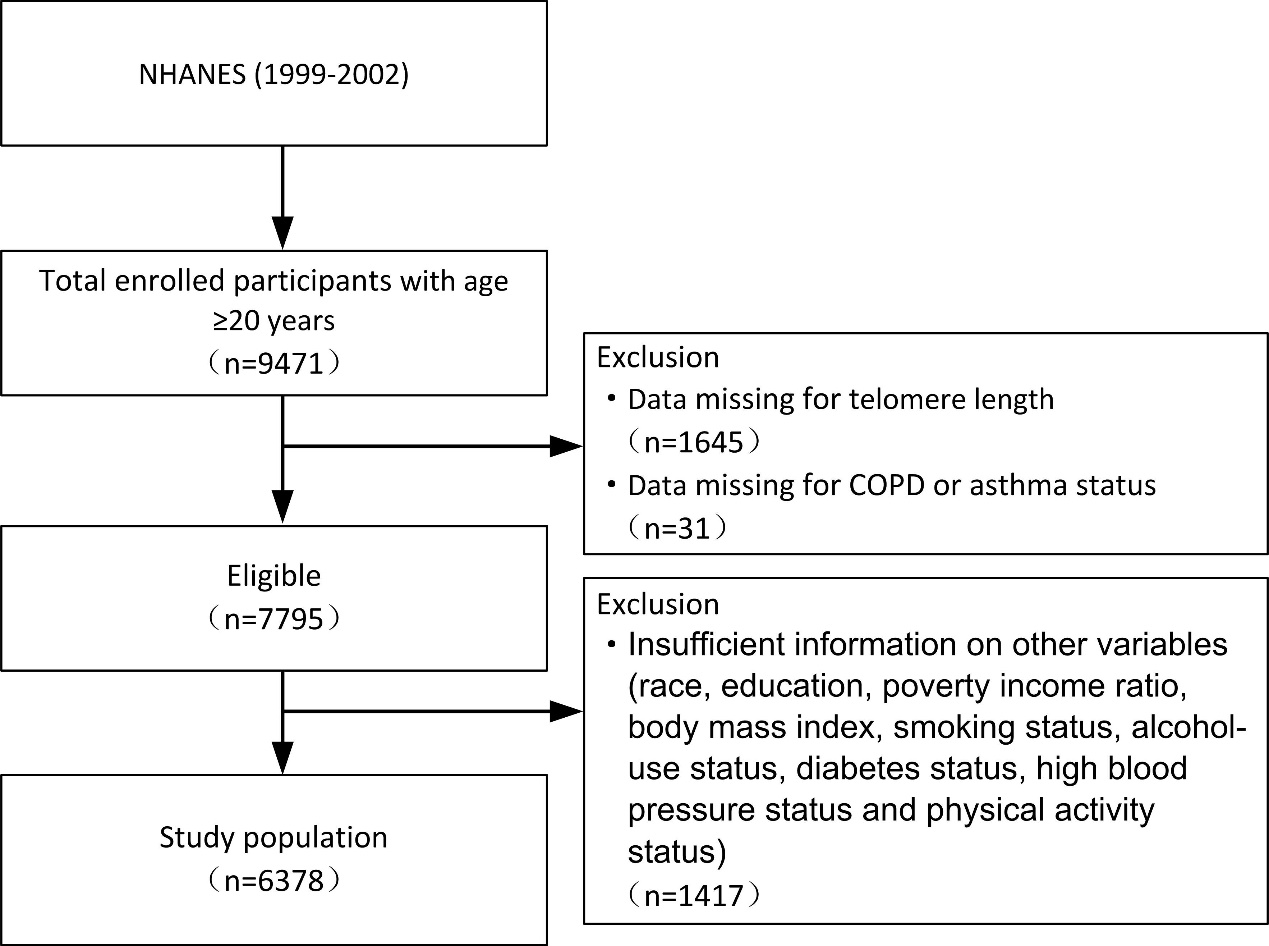


Supplementary Figure 1. Participant enrollment flowchart including the exclusion criteria-NHANES 1999-2002.


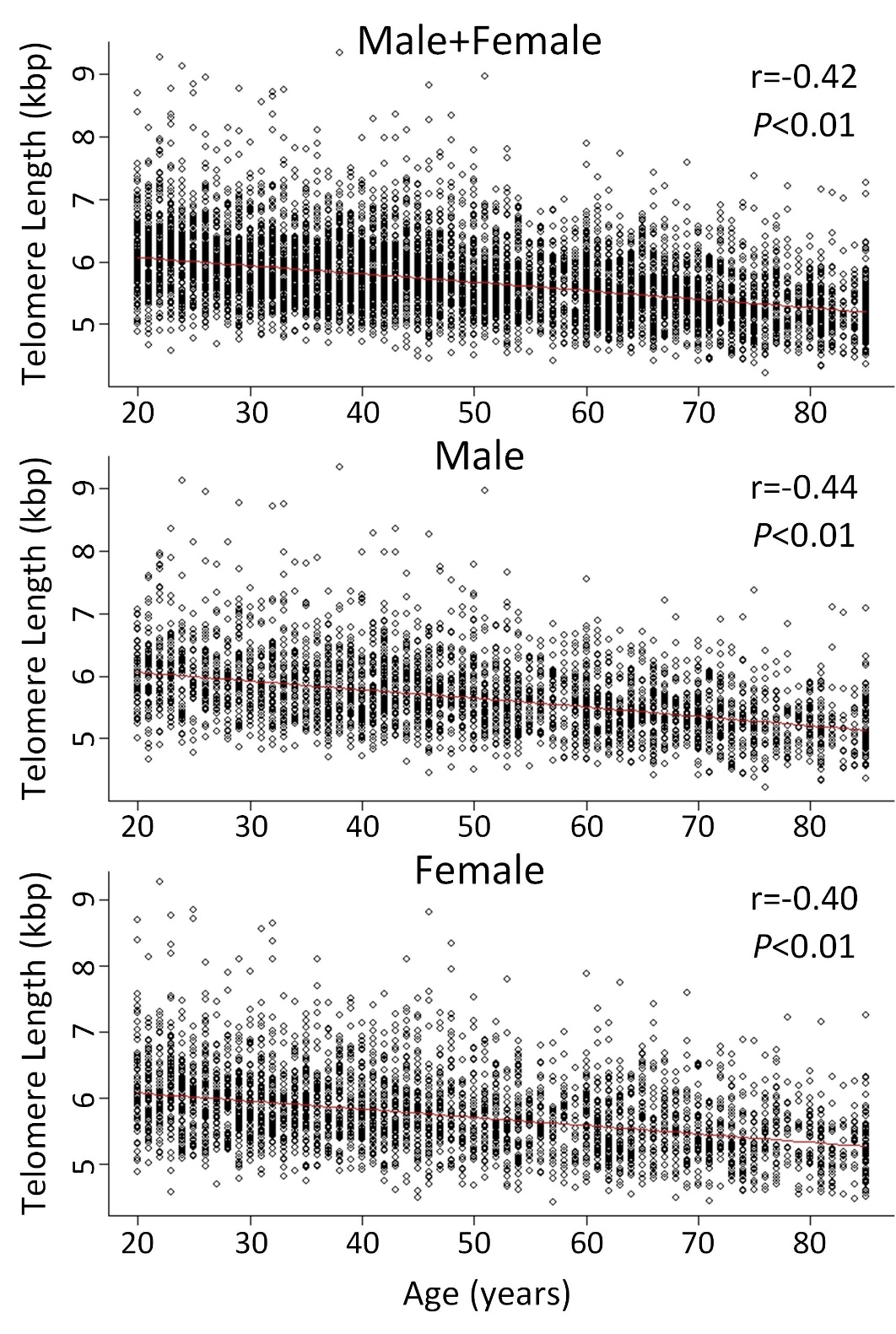


Supplementary Figure 2. Correlation between chronological age and telomere length. Three data points with telomere length > 10 kbp were removed to provide a better view of the scatter plots, and age above 85 was top coded as 85 to reduce the risk of disclosing the identity of participants in the NHANES 1999-2002.

Supplementary Table 1. Weighted smoking history of the study population by telomere length quartiles (n=6014)-NHANES 1999-2002.

|  |  | Overall | | | Telomere length | | | | | | | | | | | |  |
| --- | --- | --- | --- | --- | --- | --- | --- | --- | --- | --- | --- | --- | --- | --- | --- | --- | --- |
|  |  | | | | Quartile 1 | | | Quartile 2 | | | Quartile 3 | | | Quartile 4 | | |  |
| Variable | Status | N | % | s.e. | N | % | s.e. | N | % | s.e. | N | % | s.e. | N | % | s.e. | *P* value |
| Smoking history | Pack-years=0 | 3312 | 53.40 | 1.37 | 759 | 49.32 | 2.21 | 820 | 52.74 | 1.87 | 845 | 54.08 | 1.49 | 888 | 56.39 | 2.46 | <0.01 |
|  | 0<Pack-years≤20 | 1655 | 28.80 | 0.90 | 355 | 23.08 | 1.32 | 394 | 27.16 | 1.46 | 431 | 29.50 | 1.15 | 475 | 33.86 | 1.83 |  |
|  | 20<Pack-years≤40 | 548 | 9.85 | 0.54 | 174 | 13.81 | 1.22 | 160 | 11.26 | 1.19 | 121 | 8.87 | 0.92 | 93 | 6.56 | 0.96 |  |
|  | 40<Pack-years≤60 | 285 | 4.54 | 0.36 | 124 | 7.70 | 0.84 | 67 | 4.41 | 0.68 | 67 | 4.85 | 0.64 | 27 | 2.02 | 0.37 |  |
|  | Pack-years>60 | 214 | 3.41 | 0.34 | 92 | 6.09 | 0.83 | 62 | 4.43 | 0.70 | 39 | 2.70 | 0.48 | 21 | 1.17 | 0.36 |  |
| Abbreviations: N, Number; %, weighted percent; s.e., standard error; NHANES, National Health and Nutrition Examination Survey.  *P* value was calculated from Rao-Scott chi-square test. | | | | | | | | | | | | | | | | | |

Supplementary Table 2. Weighted smoking history of the study population by COPD status (n=6014)-NHANES 1999-2002.

|  |  | Overall | | | COPD | | | | | |  |
| --- | --- | --- | --- | --- | --- | --- | --- | --- | --- | --- | --- |
|  |  |  |  |  | Yes | | | No | | |  |
| Variable | Status | N | % | s.e. | N | % | s.e. | N | % | s.e. | *P* value |
| Smoking history | Pack-years=0 | 3312 | 53.40 | 1.37 | 169 | 37.86 | 2.47 | 3143 | 54.73 | 1.37 | <0.01 |
|  | 0<Pack-years≤20 | 1655 | 28.80 | 0.90 | 112 | 27.44 | 2.36 | 1543 | 28.92 | 0.97 |  |
|  | 20<Pack-years≤40 | 548 | 9.85 | 0.54 | 72 | 18.05 | 2.53 | 476 | 9.15 | 0.50 |  |
|  | 40<Pack-years≤60 | 285 | 4.54 | 0.36 | 35 | 7.90 | 1.68 | 250 | 4.26 | 0.35 |  |
|  | Pack-years>60 | 214 | 3.41 | 0.34 | 40 | 8.75 | 2.27 | 174 | 2.95 | 0.28 |  |
| Abbreviations: N, Number; %, weighted percent; s.e., standard error; NHANES, National Health and Nutrition Examination Survey.  *P* value was calculated from Rao-Scott chi-square test. | | | | | | | | | | | |

Supplementary Table 3. Odds ratio for COPD by smoking status (n=6378) and smoking history (n=6014)-NHANES 1999-2002.

|  | COPD | |
| --- | --- | --- |
|  | N | aOR (95% CI ) |
| Smoking status | 455 |  |
| Yes | 290 | 1.88 (1.48-2.38) |
| No | 165 | 1.00 |
| *P* |  | <0.01 |
| Smoking history | 428 |  |
| Pack-years=0 | 169 | 1.00 |
| 0<Pack-years≤20 | 112 | 1.45 (1.10-1.93) |
| 20<Pack-years≤40 | 72 | 2.65 (1.94-3.63) |
| 40<Pack-years≤60 | 35 | 2.41 (1.35-4.33) |
| Pack-years>60 | 40 | 3.61 (1.96-6.63) |
| *P* for trend |  | <0.01 |
| Model was adjusted for sex, age, age square,  education, race, poverty income ratio, body mass index,  smoking status, alcohol-use status, diabetes status,  high blood pressure status, physical activity status  and telomere length. | | |

Supplementary Table 4. Odds ratio for COPD by telomere length quartiles (n=6014)-NHANES 1999-2002.

|  |  | COPD | |  | |
| --- | --- | --- | --- | --- | --- |
| Telomere length | N | | aOR (95% CI)^a^ | | |
| Male+Female^b^ | 428 | |  | | |
| Q4 | 79 | | 1.00 | | |
| Q3 | 92 | | 0.93 (0.63-1.37) | | |
| Q2 | 103 | | 1.21 (0.87-1.68) | | |
| Q1 | 154 | | 1.60 (1.12-2.29) | | |
| *P* for trend | | | <0.01 | | |
| Male | 160 | |  | | |
| Q4 | 24 | | 1.00 | | |
| Q3 | 30 | | 0.97 (0.47-2.02) | | |
| Q2 | 41 | | 1.29 (0.75-2.21) | | |
| Q1 | 65 | | 1.76 (0.95-3.26) | | |
| *P* for trend | | | 0.05 | | |
| Female | 268 | |  | | |
| Q4 | 55 | | 1.00 | | |
| Q3 | 62 | | 0.89 (0.57-1.38) | | |
| Q2 | 62 | | 1.17 (0.76-1.80) | | |
| Q1 | 89 | | 1.49 (0.97-2.29) | | |
| *P* for trend | | | 0.04 | | |
| Abbreviations: aOR, Adjusted odds ratio; CI, confidence interval; N, Number; NHANES, National Health and Nutrition Examination Survey. | | | | |  |
| a Model was adjusted for age, age square, education, race, poverty income ratio, body mass index, smoking history, alcohol-use status, diabetes status, high blood pressure status and physical activity status. | | | | |  |
| b Sex was further adjusted. | | | | |  |

Supplementary Table 5. Mediation of 5 inflammatory factors for the associations between age and COPD.

|  | M:CRP |  | M: Fibrinogen |  | M:WBC |  | M:B-Neu |  | M:B-Eos |  |
| --- | --- | --- | --- | --- | --- | --- | --- | --- | --- | --- |
|  | β(95%CI) | *P* value | β(95%CI) | *P* value | β(95%CI) | *P* value | β(95%CI) | *P* value | β(95%CI) | *P* value |
| X→M | 0.0093 (0.0065, 0.0120) | <0.001 | 0.0040 (0.0035, 0.0045) | <0.001 | -0.0003 (-0.0009, 0.0004) | 0.4371 | 0.0010 (0.0001, 0.0019) | 0.0273 | 0.0019 (0.0005, 0.0033) | 0.0089 |
| M\|X→Y | 0.2004 (0.0975, 0.3033) | <0.001 | 0.9266 (0.3520, 1.5012) | 0.0016 | 0.7046 (0.2764, 1.1328) | 0.0013 | 0.5472 (0.2154, 0.8790) | 0.0012 | 0.1553 (-0.0509, 0.3615) | 0.1400 |
| X\|M→Y | 0.0124 (0.0032, 0.0215) | 0.0080 | 0.0102 (0.0008, 0.0195) | 0.0331 | 0.0141 (0.0050, 0.0231) | 0.0024 | 0.0133 (0.0042, 0.0224) | 0.0041 | 0.0119 (0.0026, 0.0212) | 0.0120 |
| X→M→Y | 0.0019 (0.0008, 0.0032) | - | 0.0037 (0.0014, 0.0062) | - | -0.0002 (-0.0008, 0.0003) | - | 0.0005 (0.0001, 0.0013) | - | 0.0003 (-0.0001, 0.0010) | - |
| X = age; M = mediator (log-transformed CRP, fibrinogen, WBC, B-Neu or B-Eos); Y = COPD.  Analytical process includes: 1) an effect of X on M (X→M); 2) an effect of M on Y controlling for X (M\|X→Y); 3) a direct effect of X on Y, i.e. an effect of X on Y controlling for M (X\|M→Y); and 4) an indirect effect of X on Y, i.e. an effect of X on Y mediated by M (X→M→Y). Each process was adjusted for sex, education, race, poverty income ratio, body mass index, smoking status, alcohol-use status. | | | | | | | | | | |

**Full methods of individual-level data analyses using NHANES**

**Study population of individual-level data analyses**

The NHANES is a complex survey that is designed to collect information about the health condition of the noninstitutional civilian population in the U.S. [1-4]. The survey data have been released by the National Center for Health Statistics (NCHS) every two years since 1999 and are publicly available. The NHANES has been approved by the NCHS Research Ethics Review Board. In the current study, data from two NHANES survey cycles (1999-2002) were employed to investigate the association between telomere length and COPD. Our analyses were restricted to participants aged ≥20 years. Of the 9,471 sampled persons aged ≥20 years, 7,826 (82.6%) had telomere length information, and 31 participants were further excluded due to inadequate information on COPD/asthma status. We also excluded 1,417 participants who had insufficient information for other variables, leading to a final population of 6,378 adults (Supplementary Figure 1). For the analyses involving smoking history adjustment, participants with missing data were further excluded, leading to a study population of 6,014 participants (Supplementary Tables 1-4). Participants with missing information on inflammatory biomarkers were excluded from the mediation study, and 4,011 adults were included in the analyses.

**Telomere length measurement**

Blood samples were obtained from eligible participants and stored for DNA analyses. The telomere length assay was conducted using quantitative polymerase chain reaction (PCR) at the University of California, San Francisco, and telomere length relative to standard reference DNA (T/S ratio) was recorded [5]. The T/S ratio was converted to base pairs (bp) using the formula (3,274 + 2,413 * (T/S ratio)) as suggested by the NHANES.

**COPD status**

Self-reported physician diagnosis respiratory conditions were collected in the NHANES based on a series of questions. Participants were considered to have chronic bronchitis or emphysema if they gave a positive response to the question “Has a doctor or other health professional ever told you that you had chronic bronchitis?” or “Has a doctor or other health professional ever told you that you had emphysema?”, respectively. COPD was defined by a self-reported doctor diagnosis of chronic bronchitis or emphysema [6]. Similarly, doctor-diagnosed asthma was defined by answering the question “Has a doctor or other health professional ever told you that you have asthma?”.

**Other variables**

The relationship between telomere length and COPD status was adjusted for various potential confounding factors: age in years (20–39, 40–59, 60–79 or ≥80), race (Non-Hispanic white, Non-Hispanic black or others), education (<high school, high school or >high school), poverty income ratio (PIR; <1, 1≤PIR≤median or >median, where medians were calculated based on PIR ≥1 for each of the two data cycles), body mass index (BMI; <25 kgm^-2^ or ≥25 kgm^-2^, where BMI ≥25 kgm^-2^ indicates overweight based on NIH health guidelines), smoking status (yes or no, based on the question “Have you smoked at least 100 cigarettes in your entire life?”), alcohol usage (yes or no, based on the question “In any one year, have you had at least 12 drinks of any type of alcoholic beverage?”), diabetes status (yes or no, based on the question “Have you ever been told by a doctor or health professional that you have diabetes or sugar diabetes?”), hypertension status (yes or no, based on the question “Have you ever been told by a doctor or other health professional that you had hypertension, also called high blood pressure?”) and physical activity (yes or no, participants engaging in any vigorous or moderate activities were considered active). For smoking history, pack-years were calculated based on the product of the average number of packs smoked per day (assuming 20 cigarettes per pack) and the number of years smoked [7]. Blood samples were collected and tested in accordance with standard procedures to obtain the concentrations of five inflammatory biomarkers [C-reactive protein (CRP), fibrinogen, white blood cells (WBC) count, blood neutrophil count (B-Neu) and blood eosinophil count (B-Eos)] employed in the mediation analysis.

**Statistical analysis**

Sample weight, stratification and clustering design variables were employed to account for the complex sampling design according to the NHANES Analytic and Reporting Guidelines. The weighted characteristics were calculated based on overall data and data stratified by telomere length and COPD status. The Rao-Scott chi-square test was applied to examine significant differences for various variables. In the linear regression model for analyzing the association between age and telomere length, participants with an age ≥ 85 were excluded because age above 85 was top coded as 85 to reduce the risk of disclosing the identity of participants; thus, no exact age was reported for these older adults. The association between telomere length and COPD status was studied by logistic regression to obtain crude/adjusted odds ratios and 95% confidence intervals (CIs). *P*_trend_ was also calculated for telomere length quartiles (first quartile = 5.31, second quartile/median = 5.68 and third quartile = 6.10 kbp). In the adjusted model, age was used as a continuous variable for confounder adjustments, and age square was also adjusted to account for potential nonlinearity. The dose-response relationship for telomere length and COPD status was examined by 3-knot restricted cubic splines (RCS) using a publicly available SAS macro [8], and covariate adjustment for logistic regression was applied for restricted cubic spline analysis. SAS 9.4 software (SAS Institute Inc., Cary, NC) was employed to perform statistical analyses. Correlation analysis between chronological age and telomere length was performed in R using the PerformanceAnalytics package. In the mediation analyses, the telomere length, inflammatory biomarkers, and COPD status were selected as the independent variable (X), mediator (M) and dependent variable (Y), respectively. The total effect of X on Y was decomposed into a direct effect (i.e., an effect of X on Y controlling for M) and an indirect effect (i.e., an effect of X on Y mediated by M) [9], which were calculated using the PROCESS macro developed by Hayes [10]. All types of inflammatory biomarkers were log-transformed, and age, sex, education, race, poverty income ratio, body mass index, smoking status, and alcohol-use status were adjusted as covariates.

**Reference**

1. *Centers for Disease Control and Prevention (CDC). National Center for Health Statistics (NCHS). National Health and Nutrition Examination Survey Data. Hyattsville, MD: U.S. Department of Health and Human Services, Centers for Disease Control and Prevention, 2016,* [*http://www.cdc.gov/nchs/nhanes/nhanes_questionnaires.htm*](http://www.cdc.gov/nchs/nhanes/nhanes_questionnaires.htm)*. .*

2. Mazidi, M., et al., *Telomere attrition, kidney function, and prevalent chronic kidney disease in the United States.* Oncotarget, 2017. **8**(46): p. 80175-80181.

3. Li, S. and X. Wen, *Seropositivity to herpes simplex virus type 2, but not type 1 is associated with cervical cancer: NHANES (1999-2014).* BMC Cancer, 2017. **17**(1): p. 726.

4. Li, S., et al., *Urinary Lead Concentration Is an Independent Predictor of Cancer Mortality in the U.S. General Population.* Front Oncol, 2018. **8**: p. 242.

5. Cawthon, R.M., *Telomere measurement by quantitative PCR.* Nucleic Acids Res, 2002. **30**(10): p. e47.

6. Mannino, D.M., et al., *Chronic obstructive pulmonary disease surveillance--United States, 1971-2000.* MMWR Surveill Summ, 2002. **51**(6): p. 1-16.

7. Kim, S.J., et al., *Do early onset and pack-years of smoking increase risk of type II diabetes?* BMC Public Health, 2014. **14**: p. 178.

8. Desquilbet, L. and F. Mariotti, *Dose-response analyses using restricted cubic spline functions in public health research.* Stat Med, 2010. **29**(9): p. 1037-57.

9. VanderWeele, T.J., *A three-way decomposition of a total effect into direct, indirect, and interactive effects.* Epidemiology, 2013. **24**(2): p. 224-32.

10. Hayes, A.F.L.T.D., *Introduction to mediation, moderation, and conditional process analysis : a regression-based approach.* 2018.
